# A Novel C Type CpG Oligodeoxynucleotide Exhibits Immunostimulatory Activity *In Vitro* and Enhances Antitumor Effect *In Vivo*

**DOI:** 10.3389/fphar.2020.00008

**Published:** 2020-02-06

**Authors:** Tete Li, Jing Wu, Shan Zhu, Guoxia Zang, Shuang Li, Xinping Lv, Wenjun Yue, Yuan Qiao, Jiuwei Cui, Yan Shao, Jun Zhang, Yong-Jun Liu, Jingtao Chen

**Affiliations:** ^1^ Institute of Translational Medicine, The First Hospital of Jilin University, Changchun, China; ^2^ Cancer Center, The First Hospital of Jilin University, Changchun, China; ^3^ Changchun Huapu Biotechnology Co., Ltd., Changchun, China

**Keywords:** cytosine-phosphate-guanosine dinucleotide-containing oligodeoxynucleotides, Toll-like receptor 9, plasmacytoid dendritic cells, B cells, antitumor immunotherapy

## Abstract

**Background:**

C type CpG oligodeoxynucleotides (CpG-C ODNs), possessing the features of both A type and B type CpG ODNs, exert a variety of immunostimulatory activities and have been demonstrated as an effective antitumor immunotherapy. Based on the structural characteristics, we designed 20 potential ODNs with the aim of synthesizing an optimal, novel CpG-C ODN specific to human and murine Toll-like receptor 9 (TLR9). We also sought to investigate the *in vitro* immunostimulatory and *in vivo* antitumor effects of the novel CpG-C ODN.

**Methods:**

Twenty potential CpG-C ODNs were screened for their ability to secrete interferon (IFN)-α, and interleukin (IL)-6 and tumor necrosis factor (TNF)-α production for the three most promising sequences were assayed in human peripheral blood mononuclear cells (PBMCs) by enzyme-linked immunosorbent assay (ELISA) or cytometric bead array assay. The functions of human and mouse B cells, and cytokine production in mice induced by the most promising sequence, HP06T07, were determined by flow cytometry and ELISA. Growth and morphology of tumor tissues in *in vivo* murine models inoculated with CT26 cells were analyzed by a growth inhibition assay and immunohistochemistry, respectively.

**Results:**

Among the 20 designed ODNs, HP06T07 significantly induced IFN-α, IL-6, and TNF-α secretion, and promoted B-cell activation and proliferation in a dose-dependent manner in human PBMCs and mouse splenocytes *in vitro*. Intratumoral injection of HP06T07 notably suppressed tumor growth and prolonged survival in the CT26 subcutaneous mouse model in a dose-dependent manner. HP06T07 administered nine times at 2-day intervals (I2) eradicated tumor growth at both primary and distant sites of CT26 tumors. HP06T07 restrained tumor growth by increasing the infiltration of T cells, NK cells, and plasmacytoid dendritic cells (pDCs).

**Conclusions:**

HP06T07, a novel CpG-C ODN, shows potent immunostimulatory activity *in vitro* and suppresses tumor growth in the CT26 subcutaneous mouse model.

## Introduction

Unmethylated cytosine-phosphate-guanosine dinucleotide (CpG)-containing oligodeoxynucleotides (ODNs), also known as immunostimulatory sequences (ISS), imitate the immunoenhancing activities of bacterial DNA (Krieg et al., 1995; Krieg, 2002). In addition, as ligands for Toll-like receptor 9 (TLR9), they directly activate plasmacytoid dendritic cells (pDCs) and B cells (Hemmi et al., 2000; Bauer et al., 2001).

Based on the chemical compositions, structures, and *in vitro* activities, CpG ODNs are divided structurally and functionally into three types: A, B, and C types [also known as D (CpG-A), K (CpG-B), and CpG-C types, respectively]. CpG-A ODNs, which naturally form a multimeric structure at ~20–100 nm under physiological conditions (Kerkmann et al., 2005), are characterized by a phosphodiester central CpG-containing palindromic motif, capped at the 3’-end by a phosphorothioate-modified poly G tail (Verthelyi et al., 2001). CpG-A ODNs can induce pDCs to produce large amounts of interferon (IFN)-α and tumor necrosis factor (TNF)-α, which in turn promotes higher IFN-α- and TNF-α-dependent NK cell activity (Marshall et al., 2003; Marschner et al., 2005; Marshall et al., 2006), CD8^+^ T cell activation, and cytotoxicity (Huber and Farrar, 2011). However, they weakly stimulate TLR9-dependent nuclear factor (NF)-κB signaling, and the production of pro-inflammatory cytokines such as interleukin (IL)-6 (Marshall et al., 2003; Vollmer et al., 2004).

CpG-B ODNs contain phosphorothioate backbones and encode one or more CpG dinucleotides (Verthelyi et al., 2001; Kadowaki et al., 2001). CpG-B ODNs markedly induce B-cell activation and maturation by upregulating CD40/CD80/CD86, activate B-cell proliferation, and increase the production of cytokines such as IL-6 and TNF-α, while inducing a relatively small production of IFN-α (Krieg, 2012).

CpG-C ODNs have the characteristics of both CpG-A and CpG-B ODNs (Bao et al., 2006), and contain a full phosphorothioate backbone and a stimulatory palindromic CpG-containing motif (Sharma et al., 2008). In addition, CpG-C ODNs markedly induce cytokine secretion such as IFN-α, IL-6, and TNF-α, and stimulate the activation and proliferation of B cells (Krieg et al., 1995; Krieg, 2002; Liu et al., 2011; Li et al., 2017). Such strong stimulatory features of CpG-C ODNs show promise for using as a therapeutic immunopotentiator.

CpG-C ODNs have shown potent immune-enhancing effects that require unique sequence characteristics (Vollmer et al., 2004; Liu et al., 2011; de Titta et al., 2013). For instance, ODN 2395, a typical CpG-C ODN, has two major indispensable sequence characteristics: (a) one or more TCG elements close to, or at the 5ʹ-end of the ODN, and (b) a palindromic sequence containing multiple CpG motifs at the 3ʹ-end of the ODN (Vollmer et al., 2004). In addition, the hexameric motif 5ʹ GTCGTT in ODNs has also been demonstrated as the optimal sequence for CpG-C ODNs activities such as that of ODN 2395 (Vollmer et al., 2004). However, it has also been demonstrated that CpG-C ODNs such as C274, C695, and C792 that lack this sequence, also have very potent immunoenhancing effects (Fearon et al., 2003; Marshall et al., 2004; Marshall et al., 2005). In addition, the increasing IFN-α production correlates with longer palindromes (Marshall et al., 2005; Du et al., 2007).

CpG ODNs have been demonstrated to stimulate type-I helper T cells (Th1)-biased innate and adaptive immune responses in both pre-clinical and clinical studies (Klinman, 2004; Krieg, 2012). The stimulation is mediated by initiation of B-cell proliferation and activation (Krieg, 1996; Walker et al., 1999; Hartmann et al., 2000), enhancement of cytokine secretion (Krieg et al., 1999), or promotion of NK-cell cytotoxicity (Ballas et al., 1996). CpG ODNs have received widespread attention for using as vaccine adjuvants (Shirota and Klinman, 2014) and immunotherapeutic agents to treat various infections (Nijnik, 2013), allergies, and cancers (Klinman, 2004; van Duin et al., 2006). Extensive pre-clinical and clinical studies have provided evidence that CpG ODNs are an effective treatment option for cancers, owing to their ability to initiate immune activation in the tumor microenvironment, and break immunosuppression and tolerance (Whitmore et al., 2004). In mice, some studies have demonstrated that intratumoral injections of CpG ODNs can effectively delay tumor growth by stimulating innate and adaptive responses (Sharma et al., 2008; Marabelle et al., 2014). In humans, combining intratumoral CpG ODN with radiation has been demonstrated to be efficacious in patients with cutaneous T-cell lymphoma (Kim et al., 2012) and indolent B-cell lymphoma (Brody et al., 2010). In addition, CpG ODNs have been studied in combination with other drugs to treat cancers (Krieg, 2012; Scheiermann and Klinman, 2014), especially drugs of checkpoint inhibitors such as anti-programmed cell death 1 (PD-1) antibodies (Wang S. et al., 2016; Wang C. et al., 2016). In recent years, CpG-C ODNs, owing to the potent immunostimulatory activity, have been examined for cancer treatment. For example, SD101 (CpG-C ODN) represses tumor growth and reverts the resistance of anti-PD-1 antibodies by increasing leukocyte infiltration and type I IFN-regulated gene expression in a mouse model and a phase 1b/2 clinical experiment, respectively (Wang S. et al., 2016; Ribas et al., 2016).

In this study, we designed 20 potential ODNs based on the nucleotide sequence features of CpG-C ODNs with altered CpG motifs. The immunostimulatory properties of these CpG ODNs were comprehensively investigated to target the best novel CpG-C ODN that is specific to human and murine TLR9. We detected the abilities of the novel CpG-C ODN, HP06T07, to stimulate cytokine (IFN-α, IL-6, and TNF-α) secretion, and activation and proliferation of B cells in human peripheral blood mononuclear cells (PBMCs) and mouse splenocytes *in vitro*. In addition, we confirmed the antitumor effect of HP06T07 using the *in vivo* mouse CT26 tumor model.

## Materials and Methods

### CpG ODNs

Single-stranded, phosphorothioated ODNs containing CpG sequences were synthesized by Ribo Life Science Company (Suzhou, China). ODN 1-20 (ODN 13 was renamed HP06T07) were the sequences modified based on the nucleotide sequence characteristics of CpG-C ODNs. CpG-C control (GC) was used as the negative control for the CpG-C ODN. The positive controls were ODN 2216 (CpG-A), 2006 (CpG-B), and 2395 (CpG-C). All CpG ODNs were described in **Supplementary Table 1**. All CpG ODNs were dissolved in sterile endotoxin-free water.

### Animals

Specific-pathogen-free female BALB/c and C57BL/6 mice (Vital River Laboratory Animal Technology Co., Beijing, China), between 6 and 8 weeks old, were used in the study, and were maintained in a pathogen-free animal facility at the Institute of Translational Medicine, The First Hospital, Jilin University. All animal experiments were performed in accordance with institutional guidelines and the protocols were approved by the ethics committee of the First Hospital of Jilin University, Changchun, China (approval no.: 2017-031).

### Cells and Cell Culture

Human PBMCs were isolated using Ficoll (Corning, NY, USA) density gradient centrifugation of buffy coats obtained from healthy volunteers enrolled by the First Hospital of Jilin University. All volunteers signed an informed consent for use of their data for research purposes. The protocol used was approved by the institutional ethics committee (approval no.: 2017-031). Mouse splenocytes were isolated from 6- to 8-week-old BALB/c mice and cultured in RPMI-1640 medium (Corning, NY, USA) supplemented with 10% fetal bovine serum (FBS; Clark, USA) and 1% penicillin/streptomycin (TransGen Biotech, Beijing, China). The murine CT26 colon carcinoma cells were purchased from the American Type Culture Collection (Gaining Biological; Shanghai, China). The CT26 cells were cultured in RPMI-1640 medium supplemented with 10% FBS and 1% penicillin/streptomycin. All cells were cultured at 37°C in humidified air containing 5% CO_2_.

### Detection of Cytokines Using Enzyme-Linked Immunosorbent Assay (ELISA) and Cytometric Bead Array (CBA) Assay

Human PBMCs or mouse splenocytes were cultured at 0.2–1×10^6^ cells/well in 96-well U-bottomed plates using different concentrations of CpG ODNs. After 16 h, the supernatants were harvested and assayed with the following ELISA kits: human IFN-α, mouse IL-6, mouse TNF-α (Mabtech, Sweden), and mouse IFN-α (eBioscience, Vienna, Australia). Human IL-6 and TNF-α were analyzed using CBA (BD Biosciences, San Jose, CA, USA). All kits were used according to the manufacturers’ instructions and the results obtained were expressed as picogram per milliliter (pg/ml). For the CBA assays, 50 μl of diluted samples or recombinant standards were incubated with 50 µl mixed capture beads for 1 h at 25°C. Then, 50 µl phycoerythrin-conjugated detection antibodies were added for 2 h protected from light to form the sandwich complexes. After washing the samples to remove the unbound reagents, the concentrations of the cytokines were determined using a flow cytometer (FACS Array; BD Biosciences, San Jose, USA) and the obtained data were analyzed using the FCAP Array software (BD Biosciences, San Jose, CA, USA).

### Flow Cytometry Analysis

After stimulation with or without CpG ODNs for 24 h, human PBMCs or mouse splenocytes were collected, pre-incubated with anti-mouse CD16/32 antibodies (BD Biosciences, San Jose, CA, USA), and the dead cells were excluded using an aqua dead cell staining kit (Invitrogen, San Diego, CA, USA). The cells were stained with the following antibodies: anti-human or anti-mouse CD45, CD3, CD19, CD80, and CD86 (BD Biosciences, San Jose, USA); incubated with the respective antibodies for 25 min at 4°C; and then washed twice. After performing FACS using LSRFortessa™ cytometer (BD Biosciences, San Jose, CA, USA), the results were analyzed using the FlowJo software (BD Biosciences, San Jose, CA, USA).

### Cell Proliferation Assays

Human PBMCs or mouse splenocytes (5 × 10^5^ cells) were suspended in phosphate-buffered saline (PBS) and then stained with 5-(and 6-) carboxyfluorescein diacetate succinimidyl ester (CFSE; Invitrogen, San Diego, CA, USA) at 37°C for 7 min, protected from light. Pre-cooled RPMI-1640 medium containing FBS was then added to the cells to stop the staining process. After three rounds of washing, cells were incubated with or without CpG ODNs at 37°C for 72 h, and harvested for staining with other antibodies. All flow cytometry data were acquired using the LSRFortessa™ cytometer and were analyzed using the FlowJo software. Decreased CFSE content indicated proliferating cells.

### Syngeneic Mouse Models

For the CT26 mouse model, 2 × 10^5^ CT26 tumor cells were injected subcutaneously into the right hind flank of the 6- to 8-week-old BALB/c mice on day 0 and 1 × 10^5^ CT26 cells injected into the left side on day 4. When tumor sizes reached a maximum of 0.6–0.8 cm in diameter, 50 μl HP06T07, GC or PBS was injected into the right side of the tumors. Tumor sizes on both sides of mice were monitored using a digital caliper (AIRAJ, China) every 2–3 days, and the tumor volumes were calculated using the formula: volume (mm^3^) = (length × width × width)/2. When the tumor volumes exceeded 3,000 mm^3^, mice were euthanized.

### Immunohistochemistry

Mice were euthanized on day 28, and tumors on the right and left sides were harvested, fixed in 4% paraformaldehyde for 24 h, and paraffin-embedded for immunohistochemistry using a method reported previously (Gur et al., 2011). Paraffin-embedded spleen sections were stained with rabbit anti-mouse CD3ϵ (99940S; 1:150; Cell Signaling Technology, Danvers, MA, USA), CD19 (90176S; 1:800; Cell Signaling Technology, Danvers, MA, USA), NCR1 (ab214468; 1:600; Abcam, Cambridge, MA, USA), and rat anti-mouse PDCA-1 (DDX0390P-100; 1:100; Novus biologicals, Littleton, CO, USA) antibodies overnight, washed with Tris-buffered saline, and then incubated with goat anti-rat/rabbit (Fuzhou Maxim Biotechnology Development Co., Ltd., Fuzhou, China) and 3,3’-diaminobenzidine (DAB) substrate (Fuzhou Maxim Biotechnology Development Co., Ltd., Fuzhou, China). Images were captured using a light microscope (BX51N-34-FL-1-D, Olympus Corporation, Tokyo, Japan) and processed by CellSens Dimension software (Universal Imaging).

### Statistical Analysis

Data were analyzed using GraphPad Prism software (San Diego, CA, USA) and expressed as means ± standard error of the mean (SEM). Log-rank test was performed to compare survival curves between groups. Statistical significance of the differences between the experimental groups was determined using the Student’s t-test or two-way analysis of variance (ANOVA), followed by Bonferroni test for multiple comparisons. P values < 0.05 were considered significant (^*^P < 0.05, ^**^P < 0.01, ^***^P < 0.001, and ^****^p < 0.0001).

## Results

### The Production of IFN-α, IL-6, and TNF-α by Human PBMCs Is Effectively Induced by CpG-C ODNs

**Figure1:**
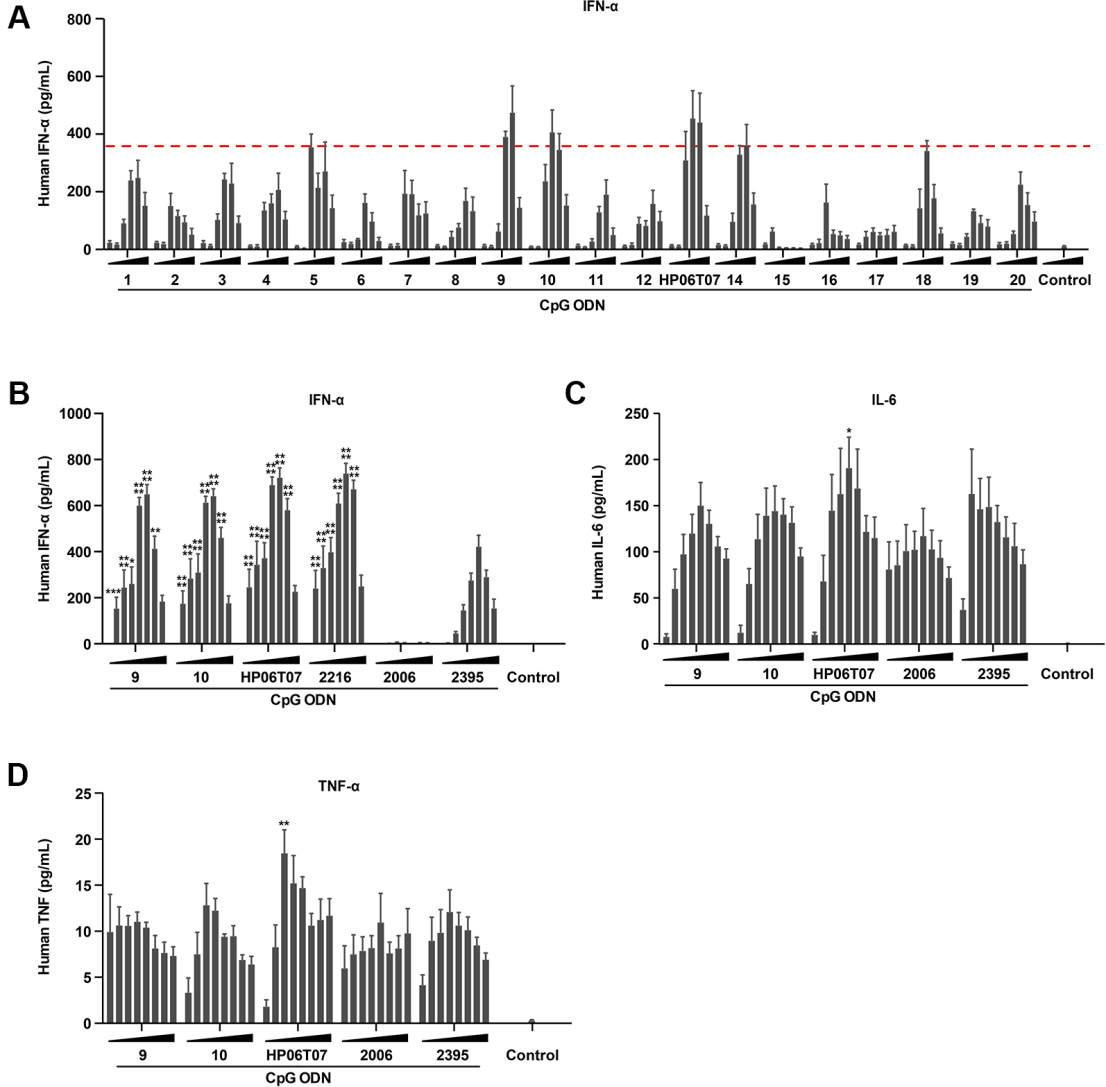
The production of IFN-α, IL-6, and TNF-α by human PBMCs is effectively induced by CpG-C ODNs. **(A)** Human PBMCs (2 × 10^5^) were cultured with or without the 20 designed CpG-C ODNs at different concentrations (0.01, 0.03, 0.1, 0.3, 1, and 3 μM) for 16 h. Supernatants were harvested and assayed for IFN-α using ELISA. **(B**–**D)** Human PBMCs (2 × 10^5^) were incubated with or without ODN 9, 10, 13 (the principal ODNs that renamed HP06T07), 2216, 2006, and 2395 at different concentrations (0.025, 0.05, 0.1, 0.2, 0.4, 0.8, 1.6, and 3.2 μM) for 16 h. Supernatants were harvested and assayed for IFN-α **(B)** using ELISA, and IL-6 **(C)** and TNF-α **(D)** *via* CBA. All data are presented as means ± SEM of three technical replicates from two donors per group. Statistical significance of differences between ODN 2395 and other treated groups were determined (^*^P < 0.05, ^**^P < 0.01, ^***^P < 0.001, and ^****^P < 0.0001).

CpG-C ODNs induce IFN-α production by pDCs (Marshall et al., 2003). ELISA results showed that ODN 9, ODN 10, and ODN 13 (the principal ODNs that renamed HP06T07) markedly induced IFN-α in a dose-dependent manner, and the induced IFN-α peaked at a CpG ODN concentration of 0.33 or 1 μM. Other CpG ODNs also stimulated IFN-α production to a certain degree, but their effects were weaker than those of ODN 9, ODN 10, and HP06T07 (**Figure 1A** and **Table S1**).

The cytokine-inducing activities of these three CpG ODNs were evaluated using ELISA or CBA with the typical CpG-C ODN, ODN 2395, as the positive control to evaluate cytokine production. ODN 2006, a CpG-B ODN, was used as the negative control for IFN-α secretion, and as a positive control for IL-6 and TNF-α production. The results showed that ODN 2395 markedly induced IFN-α, IL-6, and TNF-α production, while ODN 2006 effectively induced IL-6 and TNF-α; no obvious IFN-α secretion was observed as mentioned above. Compared to ODN 9, ODN 10, and the controls, ODN 2006 and ODN 2395, the most upregulation of IFN-α, IL-6, and TNF-α was caused by HP06T07 (**Figures 1B–D**). Owing to the potent cytokine induction by HP06T07, it was used in subsequent experiments to confirm its function *in vitro* and *in vivo*.

### Activation and Proliferation of Human B Cells Are Enhanced by HP06T07

**Figure 2:**
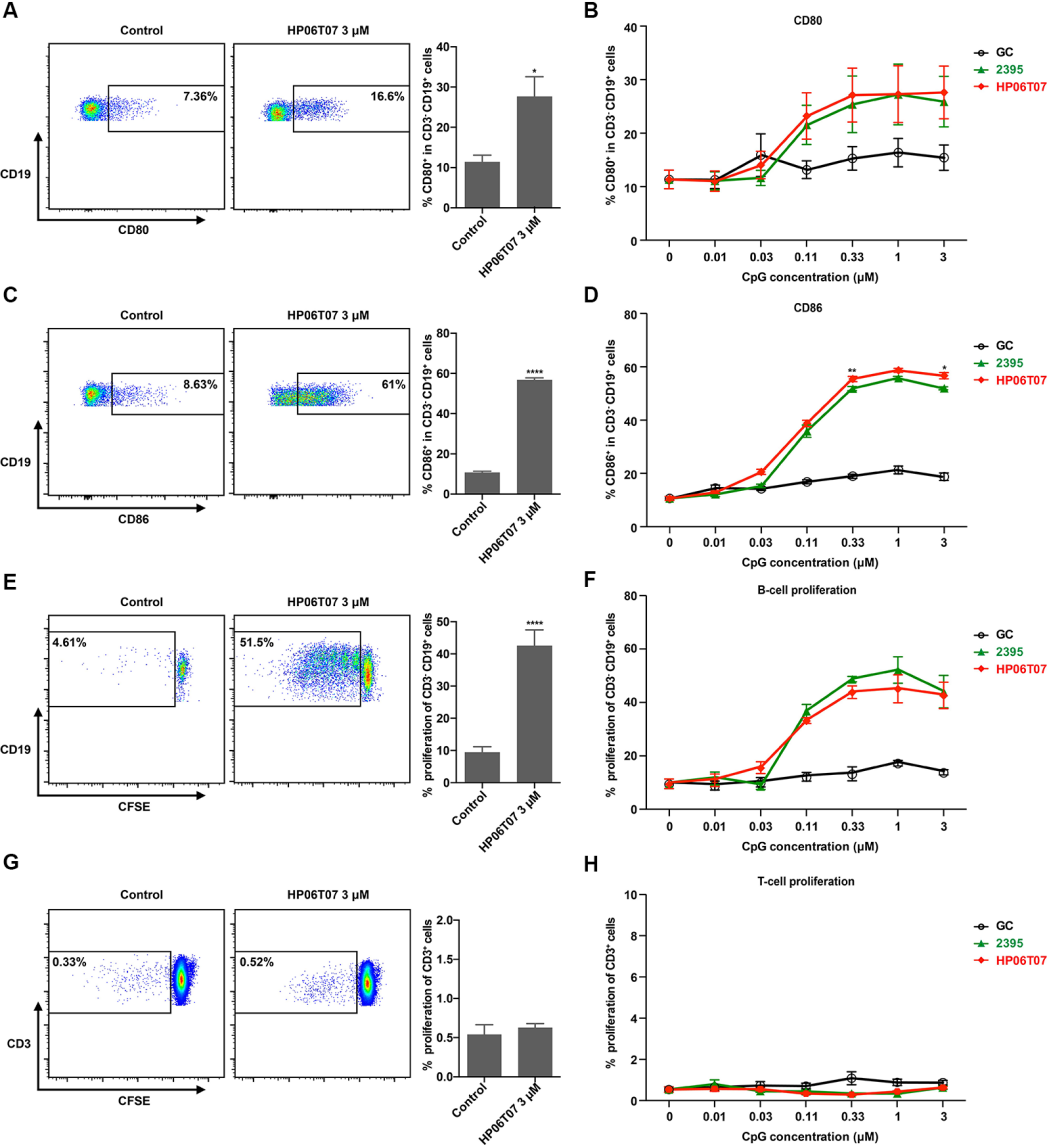
Activation and proliferation of human B cells are enhanced by HP06T07. Human PBMCs (5 × 10^5^) cultured with or without HP06T07 (red), ODN 2395 (green), and negative control GC (black) at different concentrations (0.01, 0.03, 0.1, 0.3, 1, and 3 μM) for 24 h. Cells were collected, and the dead cells were excluded using an aqua dead cell stain kit. Cells were then stained with CD3, CD19, CD80, and CD86. Representative plots and histograms of CD80 **(A)** and CD86 **(C)** expression analyzed using flow cytometry (gated on CD3^-^ CD19^+^ B cells). **(B, D)** Expression of CD80 and CD86 on CD3^−^ CD19^+^ B cells. **(E–H)** Human PBMCs (5 × 10^5^) incorporated with CFSE and cultured with or without HP06T07, ODN 2395, and the negative control GC at different concentrations (0.01, 0.03, 0.1, 0.3, 1, and 3 μM) for 3 days. CD19^+^ B cell **(E, F)** and CD3^+^ T cell **(G, H)** proliferation was measured by decreasing the CFSE content. All data are presented as means ± SEM of three technical replicates from two donors per group. Statistical significance of differences between HP06T07 and ODN 2395 groups were determined (^*^P < 0.05, ^**^P < 0.01, and ^****^P < 0.0001).

CpG-C ODNs not only induce the production of cytokines, but promote B-cell activation and proliferation (Marshall et al., 2003). Flow cytometry analysis revealed that GC had no obvious effect on CD80 and CD86 expression in B cells. ODN 2395 significantly promoted CD80 and CD86 expression in B cells to increase their activation and maturation in a dose-dependent manner. HP06T07 also significantly promoted CD80 and CD86 expression in B cells in a dose-dependent manner (**Figures 2A–D**). In addition, the proliferation of B cells was determined using CFSE incorporation after stimulation for 3 days. Similar to the activation and maturation of B cells, HP06T07 and ODN 2395 increased the proliferation of B cells in a dose-dependent manner, whereas GC had no effect on B-cell proliferation (**Figures 2E, F**). All CpG ODNs had no direct effect on the proliferation of T cells (**Figures 2G, H**).

In addition, all CpG ODNs had no effect on IFN-α, IL-6, and TNF-α production (**Figures S1C–E**) and CD80 and CD86 expression (**Figures S1F, G**) in human monocyte-derived dendritic cells (MDDCs; **Figures S1A, B**) that did not express TLR9, suggesting that HP06T07 was specific to human TLR9.

### Secretion of Cytokines and Functions of B Cells in Mouse Splenocytes Are Effectively Increased by HP06T07

**Figure 3:**
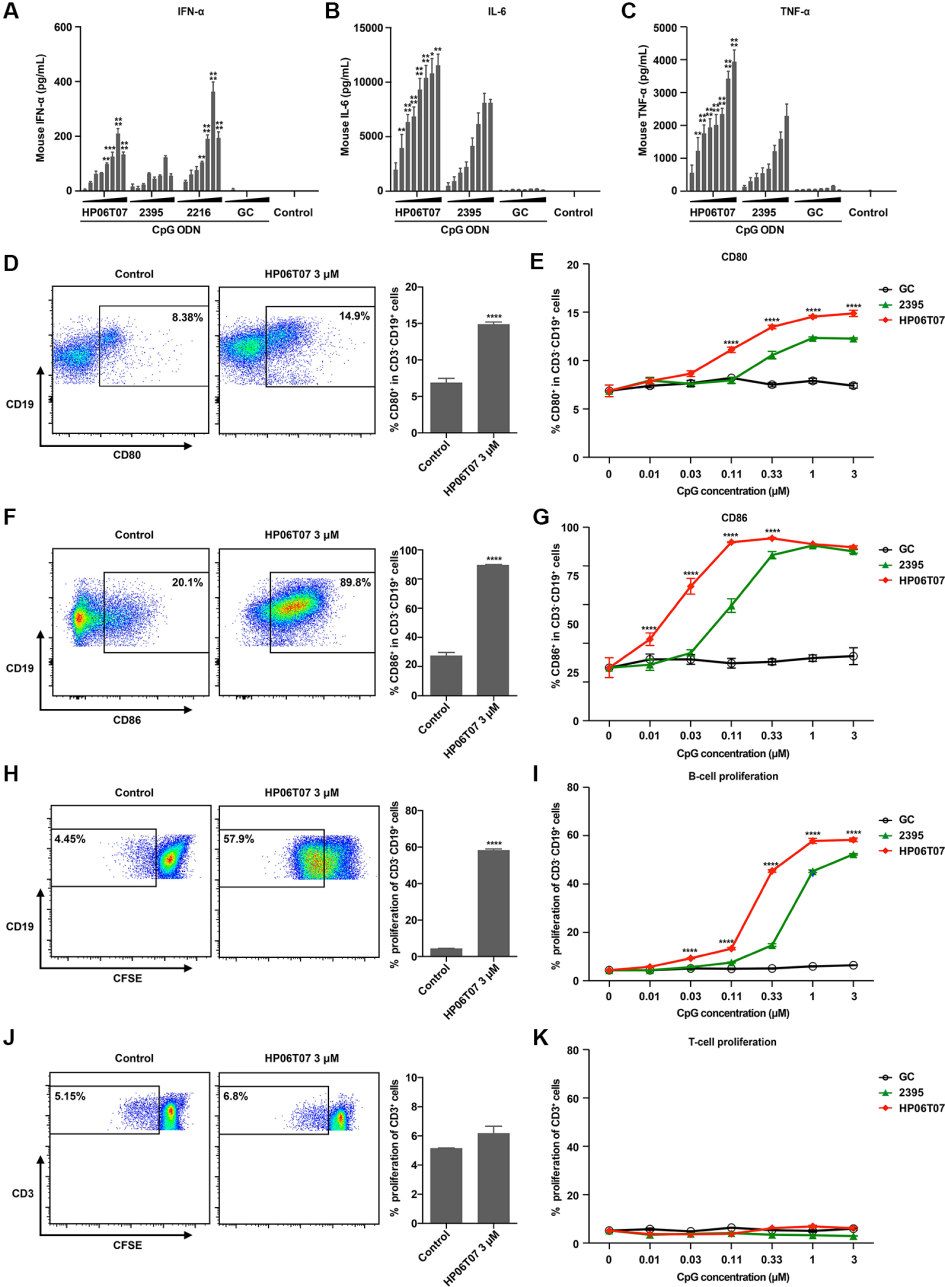
Secretion of cytokines and functions of B cells in mouse splenocytes are effectively increased by HP06T07. **(A)** Mouse splenocytes (1 × 10^6^) were cultured with or without HP06T07 (red), ODN 2395 (green), negative control GC (black), and ODN 2216, at different concentrations (0.025, 0.05, 0.1, 0.2, 0.4, 0.8, 1.6, and 3.2 μM) for 16 h. Supernatants were harvested and assayed for IFN-α *via* ELISA. **(B–C)** Mouse splenocytes (5 × 10^5^) were stimulated with or without HP06T07, ODN 2395 and negative control GC at different concentrations (0.025, 0.05, 0.1, 0.2, 0.4, 0.8, 1.6, and 3.2 μM) for 16 h. Supernatants were harvested and assayed for IL-6 **(B)** and TNF-α **(C)** using ELISA. **(D–G)** Mouse splenocytes (5 ×10^5^) were cultured with or without HP06T07, ODN 2395, and negative control GC at different concentrations (0.01, 0.03, 0.1, 0.3, 1, and 3 μM) for 24 h. Dead cells were excluded using the aqua dead stain kit and cells were stained with CD3, CD19, CD80, and CD86. Representative plots and histograms of CD80 **(D)** and CD86 **(F)** expression were analyzed using flow cytometry (gated on CD3^−^ CD19^+^ B cells). **(E, G)** Expression of CD80 and CD86 on CD3^−^ CD19^+^ B cells. **(H–K)** Mouse splenocytes (5 × 10^5^) were incorporated with CFSE and cultured with or without HP06T07, ODN 2395, and negative control GC at different concentrations (0.01, 0.03, 0.1, 0.3, 1, and 3 μM) for 3 days. CD19^+^ B cell **(H, I)** and CD3^+^ T cell **(J, K)** proliferation was measured by decreasing CFSE content. All data are presented as means ± SEM of two to three technical replicates from two independent experiments per group. Statistical significance of differences between HP06T07 or ODN 2216 and ODN 2395 groups were determined (**P < 0.01, ***P < 0.001, and ****P < 0.0001).

HP06T07 markedly stimulated human PBMCs to secrete IFN-α, IL-6, and TNF-α, and enhanced B-cell activation and proliferation. To confirm the immune-enhancing function of HP06T07 in mice, similar studies were performed. In evaluating the production of cytokines, ELISA revealed that similar to the effect on human PBMCs, HP06T07 induced higher IFN-α, IL-6, and TNF-α secretion than ODN 2395, whereas GC did not induce the secretion of these cytokines (**Figures 3A–C**).

We additionally detected the activation and proliferation of B cells in mouse splenocytes stimulated by CpG ODNs. Mouse splenocytes were stimulated with or without HP06T07, ODN 2395, and the negative control GC; all were triple diluted (0.01, 0.03, 0.11, 0.33, 1, and 3 μM). As observed with human B cells, ODN 2395 induced B cells to express CD80 and CD86 (**Figures 3D–G**), and promoted B-cell proliferation (**Figures 3H, I**). GC had no effect on B-cell activities. HP06T07 enhanced B-cell activation and proliferation with higher values observed than ODN 2395 (**Figures 3D–I**). Furthermore, all CpG ODNs did not affect the proliferation of T cells (**Figures 3J, K**).

We next investigated mouse TLR9 (mTLR9) activation by HP06T07 using HEK-Blue™-mTLR9 cells (**Figure S1H**) and the parental cell line HEK-Blue™ Null1 cells (**Figure S1I**). HP06T07 more markedly augmented the activation of mTLR9 at a low concentration than ODN2395 did, while GC had no effect in HEK-Blue™-mTLR9 cells (**Figure S1H**). All CpG ODNs had no effect in HEK-Blue™ Null1 cells that did not express mTLR9 (**Figures S1I–K**). These results suggested that the novel CpG-C ODN, HP06T07, was specific to mTLR9.

### *In Vivo* Treatment With Different Doses and Administration Regimen of HP06T07 Restrains CT26 Tumor Growth

**Figure 4:**
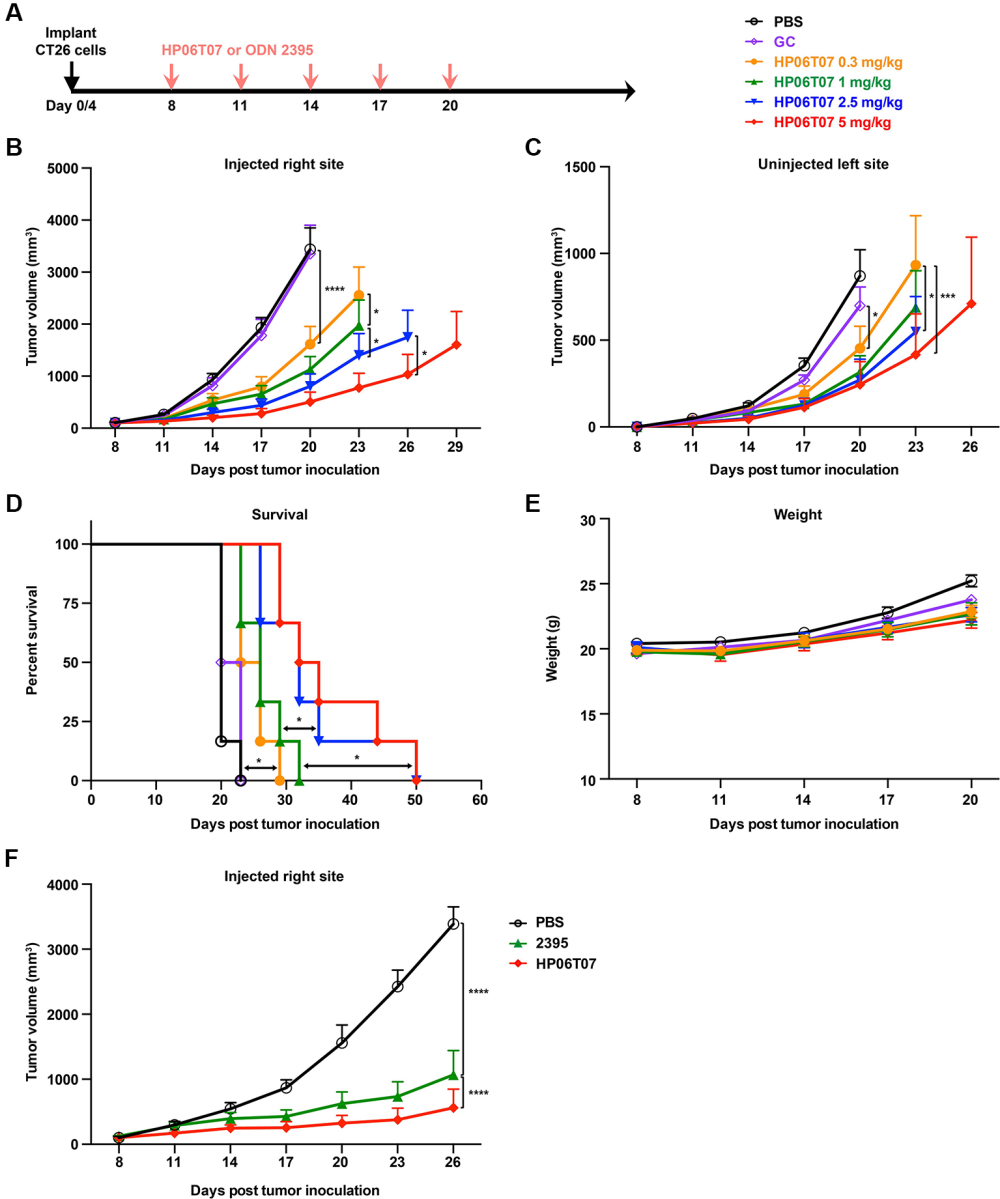
*In vivo* treatment with different doses of HP06T07 exhibits diverse immunotherapeutic effect on CT26 tumors. **(A)** Experimental protocol for HP06T07 treatment with different doses or ODN 2395 treatment. CT26 (2 × 10^5^) cells were implanted subcutaneously into right flanks of 6- to 8-week-old BALB/c mice on day 0, and the CT26 (1 × 10^5^) cells injected into the left side on day 4. When tumor sizes reached a maximum of 0.6–0.8 cm in diameter (day 8), different doses of HP06T07 (0.3, 1, 2.5, and 5 mg/kg; yellow, green, blue, and red, respectively) or GC (2.5 mg/kg; purple) were intratumorally injected into the right side of tumors on day 8, 11, 14, 17, and 20. Tumor sizes on both sides of mice were monitored using a digital caliper, every 3 days. **(B)** Tumor volumes of injected right site of tumors over time. **(C)** Tumor volumes of uninjected left site of tumors over time. **(D)** Overall survival over time. **(E)** Mouse weights over time. **(F)** Tumor volumes of injected right site of tumors in mouse treated with or without 5 mg/kg HP06T07 (red) or ODN2395 (green). All data are means ± SEM (n = 6/group). Statistical significance of differences was determined (^*^P < 0.05, ^*^P < 0.01, ***P < 0.001, and ****P < 0.0001).

The above results showed that the HP06T07 designed as a CpG-C ODN clearly promoted B-cell functions and enhanced the secretion of cytokines including IFN-α, IL-6, and TNF-α. To further evaluate whether intratumoral treatment with HP06T07 suppresses tumor growth, and confirm the optimal dose, CT26 cells were implanted in both flanks of BALB/c mice. Firstly, the CT26 tumor-bearing mice were divided into six groups and intratumorally injected with PBS, the CpG-C negative control GC, and four doses of HP06T07 (0.3, 1, 2.5, and 5 mg/kg) in the right flank on day 8, 11, 14, 17, and 20 (**Figure 4A**). Tumor growth and mouse weights were then monitored (**Figures 4B–E**). HP06T07 significantly suppressed tumor growth in a dose-dependent manner on the right injected and left uninjected sites. In addition, HP06T07 (5 mg/kg) showed the most obvious immunotherapeutic effect (**Figures 4B, C**). The survival study illustrated that HP06T07 improved the survival of mice bearing CT26 tumors (**Figure 4D**). HP06T07 did not reduce the weight of the mice, suggesting that HP06T07 does not induce major side effects (**Figure 4E**). Furthermore, HP06T07 (5 mg/kg) significantly suppressed tumor growth more than ODN 2395 (5 mg/kg) treatment did (**Figure 4F**).

**Figure 5:**
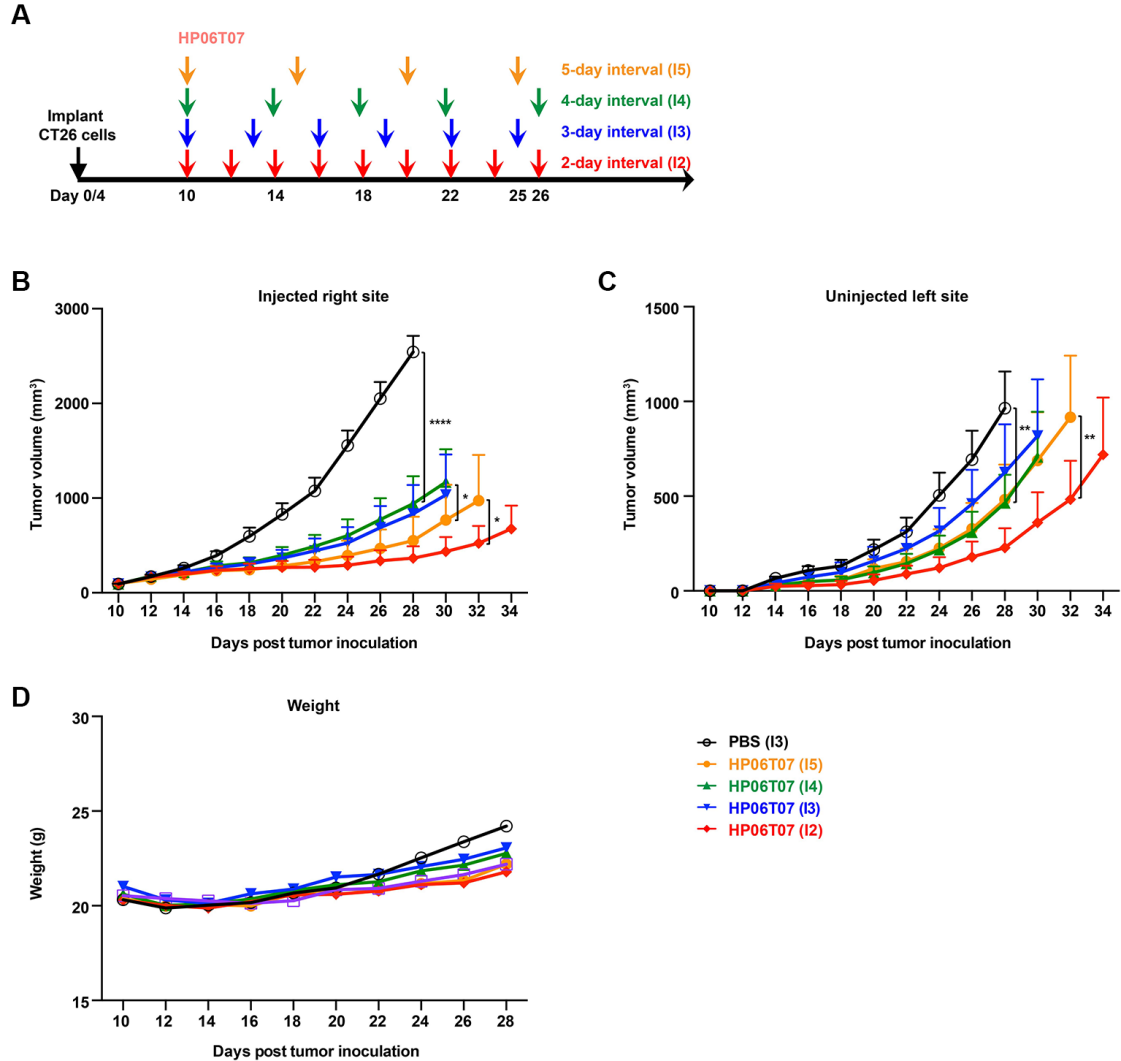
*In vivo* treatment with different administration regimens of HP06T07 suppresses tumor growth. **(A)** Experimental protocol of HP06T07 treatment with different administration regimens. CT26 (2 × 10^5^) cells were implanted subcutaneously into the right flank of 6- to 8-week-old BALB/c mice on day 0, and on day 4 in left flank using the CT26 (1 × 10^5^) cells. When tumor sizes reached a maximum of 0.6–0.8 cm in diameter (day 10), 3 mg/kg HP06T07 was intratumorally injected into right side of tumors at 50 μl, and was administered consecutively four times at 5-day intervals (days 10, 15, 20, and 25; I5; yellow), five times at 4-day intervals (day 10, 14, 18, 22, and 26; I4; green), six times at 3-day intervals (day 10, 13, 16, 19, 22, and 25; I3; blue) or nine times at 2-day intervals (day 10, 12, 14, 16, 18, 20, 22, 24, and 26; I2; red). Tumor sizes on both sides of mice were monitored using digital calipers, every 2 days. Tumor growth on injected right **(B)** and uninjected left **(C)** sites was monitored. **(D)** Mouse weights over time. All data are means ± SEM (n = 8/group). Statistical significance of differences was determined (^*^P < 0.05, ^**^P < 0.001, and ^****^P < 0.0001).

In addition, bilateral CT26 tumor models were treated with 3 mg/kg HP06T07 at different administration intervals and times (**Figure 5A**), which repressed tumor progression to some extent. The consecutive administration of HP06T07 nine times at 2-day intervals (I2), had the best antitumor effect in delaying CT26 tumor growth at both primary and distant sites (**Figures 5B, C**). HP06T07 administered four times at 5-day intervals (I5) also exhibited antitumor effect. HP06T07 administered six times at 3-day intervals (I3), and five times at 6-day intervals (I4) displayed similar antitumor effect, but weaker than those of I2 and I5 (**Figures 5B, C**). Overall, HP06T07 did not decrease mouse weights (**Figure 5D**).

### Treatment With HP06T07 Induces Accumulation of T Cells, NK Cells, and pDCs

**Figure 6:**
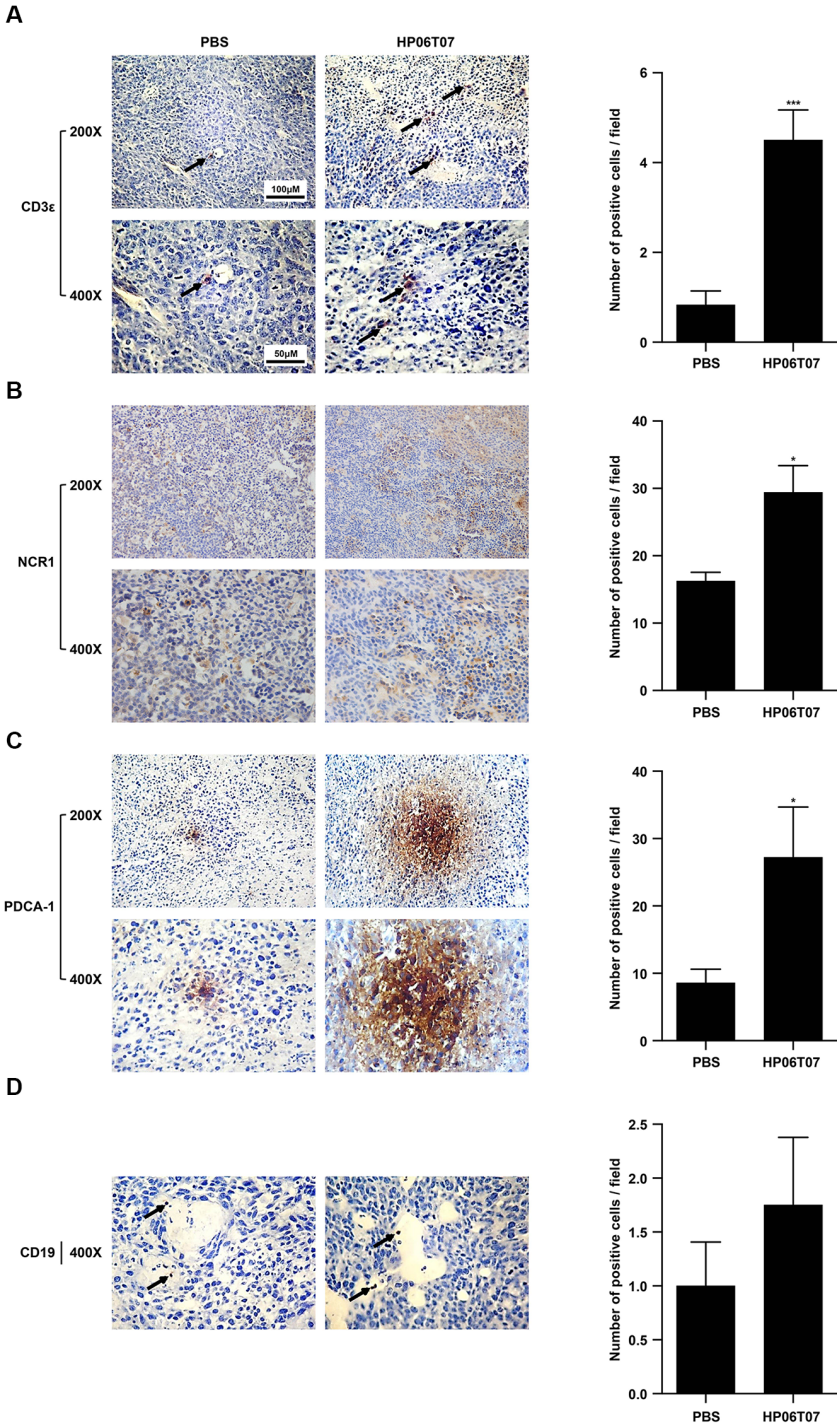
Treatment with HP06T07 induces accumulation of T cells, NK cells, and pDCs. Three mg/kg HP06T07 or PBS was intratumorally injected into right sides of tumors at 50 μl and was administered consecutively six times at 3-day intervals (day 10, 13, 16, 19, 22, and 25; I3). Two days after last treatment, tumors on the right sides of mice were harvested, embedded with paraffin, and stained with anti-mouse CD3ϵ **(A)**, NK cell marker NCR1 **(B)**, pDCs marker PDCA-1 **(C)**, and CD19 **(D)**. Positive cells were detected with alkaline phosphatase-conjugated goat anti-rat/rabbit IgG according to the manufacturer’s instructions. Representative images from one of three HP06T07- or PBS-treated mice are shown. Arrows indicate infiltration of immune cells. Original magnification, 200× and 400×. Histogram results were expressed as mean ± SEM of positive cells/field evaluated at 400× images (n = 4–6/group). Statistical significance of differences was determined (*P < 0.05 and ^***^P < 0.001).

To characterize the antitumor effects of HP06T07 on tumor-infiltrating immune cells, tumors of mice treated with HP06T07 or PBS administered six times at 3-day intervals (I3) were harvested 2 days after the last treatment. Immunohistochemistry of tumor sections from right side of HP06T07-treated mice showed CD3^+^ cells (**Figure 6A**), whereas the control mice showed few CD3^+^ cells. In addition, HP06T07 promoted the infiltration of NCR1^+^ (**Figure 6B**) and PDCA-1^+^ (**Figure 6C**) cells, compared to the PBS-treated group. No significant changes in CD19^+^, a B-cell specific surface antigen, were observed in tumors between the HP06T07- and PBS-treated groups (**Figure 6D**). These results demonstrated that HP06T07 increased the infiltration of T cells, NK cells, and pDCs.

## Discussion

In this study, we aimed to screen novel CpG-C ODNs developed with proprietary intellectual property rights. To this end, *in vitro* and *in vivo* experimental studies were performed to evaluate the immunostimulatory properties of the novel CpG-C ODN that is specific for humans and mice, from the 20 potential ODNs designed based on the nucleotide sequence characteristics of CpG-C ODNs.

The data clearly indicate that HP06T07 is an effective CpG-C ODN. CpG-C ODNs markedly induce IFN-α, IL-6, and TNF-α secretion and stimulate B cells (Marshall et al., 2003). Firstly, the designed HP06T07 significantly induced IFN-α secretion from human PBMCs and mouse splenocytes compared to the control formulation (**Figures 1A, B** and **3A**). Secondly, HP06T07 enhanced IL-6 and TNF-α production in human and mouse cells *in vitro* (**Figures 1C, D** and **3B, C**). HP06T07 also markedly increased CD80 and CD86 expression in human and murine B cells (**Figures 2A–D** and **3D–G**), and promoted B cell proliferation (**Figures 2E, F** and **3H, I**). Thus, the data obtained clearly demonstrated that HP06T07 is an effective immunostimulatory human and murine CpG-C ODN.

The detection of cytokine production is a conventional method for the screening of CpG ODNs. In this study, IFN-α production was first checked to screen 20 potential CpG-C ODNs except CpG-B ODNs. We found that ODN 9, ODN 10, and HP06T07 significantly induced IFN-α secretion in human PBMCs. IFN-α has many biological functions including promoting the proliferation of Th1 (Belardelli, 1995), increasing the tumor-specific cytolytic T cell activity (von Hoegen et al., 1990), and suppressing tumor growth and tumor angiogenesis (Okada et al., 2001). Therefore, these three most promising ODNs were selected for a further study to detect IL-6 and TNF-α production. We found that HP06T07 markedly promoted IL-6 and TNF-α production. Thus, HP06T07 was deemed the most promising CpG-C ODN. In addition, HP06T07 enhanced the functions of B cells in human and mouse, and increased cytokine production in mouse. Thus, HP06T07 is an effective CpG-C ODN specific for humans and mice.

HP06T07, a 29-nucleotide phosphorothioate oligodeoxynucleotide, significantly induced the secretion of cytokines in human PBMCs and mouse splenocytes *in vitro*. However, certain cytokines could be produced by several immune cells. For example, IFN-α is predominantly secreted by pDCs (Liu, 2005); however, other immune cells such as macrophages and T cells also produce IFN-α. TNF-α is produced by pDCs, B cells, and monocytes/macrophages, among other cells (Campbell et al., 2009). In the present study, we determined the concentration of the cytokines stimulated by HP06T07 in human PBMCs and mouse splenocytes; however, we were unable to identify the cells induced by CpG ODNs. Thus, flow cytometry or sorted pDCs and B cells should be used as more precise methods to further determine the cell-specific activation of HP06T07 in future studies.

In our *in vivo* experiments, tumor growth in CT26 subcutaneous model treated with different concentrations of HP06T07 delivered to the tumor was notably suppressed in a dose-dependent manner (**Figure 4**). These results indicated that HP06T07 owing to its powerful immunostimulatory effect, has efficient antitumor activity and is expected to be one of the most potent monotherapies for cancers. The effects of the intratumoral injection aligns with previous reports that *in situ* vaccination with CpG-C ODN significantly inhibited the occurrence and development of tumors (Sato-Kaneko et al., 2017; Sagiv-Barfi et al., 2018). We also investigated the antitumor effect of different administration regimens (intervals and times) of HP06T07 (**Figure 5**). We demonstrated that HP06T07 administered at I2 and I5 had the best antitumor effect by delaying CT26 tumor growth on both right and left sites (**Figure 5**). Therefore, intensive monotherapy with HP06T07 either continuously or at regular intervals achieved superior therapeutic effects.

*In vivo* treatment with different doses and administration regimen of HP06T07 did not decrease mouse weights. However, the HP06T07-treated groups appeared to experience slightly less weight gain than the PBS-treated group. The reason might be that HP06T07 significantly suppressed tumor growth to keep tumors at small volumes, while the tumor in the PBS-treated group could not be controlled and thus, kept increasing. Consequently, the overall mouse weights (pure mouse weights and tumor weights) in the PBS-treated group were obviously increased especially when tumor volumes exceeded 1,000 mm^3^.

Intratumoral injection of HP06T07 enhanced the infiltration of CD3^+^ T cells, NCR1^+^ NK cells, and PDCA-1^+^ pDCs as shown by immunohistochemistry (**Figure 6**). CD3 is commonly used as a T-cell-specific marker. NKp46, a major killer receptor, is expressed exclusively by NK and NK-like cells for which an orthologous protein, NCR1, has been found in mice (Gur et al., 2010). Thus, NCR1 is considered as the most specific mouse NK cell marker (Gur et al., 2011). B220 (CD45R) and PDCA-1 (BST2) are important specific phenotype markers of pDCs (Narendra et al., 2014; Kostarnoy et al., 2017). However, B220 was expressed not only on pDCs, but also on B cells and activated T cells (Simon et al., 2018). Therefore, PDCA-1 was considered the more accurate and specific marker. CD19 is usually used as a B-cell marker. In our studies, CD19^+^ cells were few and scattered, and no significant difference occurred between HP06T07-treated and PBS-treated groups. In our *in vitro* experiments, however, HP06T07 significantly promoted B-cell proliferation. A possible reason to explain this contradiction might be that the B cells were only a small population of immune cells in the subcutaneous tumor tissues, making them difficult to detect using immunohistochemistry. Flow cytometry analysis to detect the infiltrations and functions of immune cells might solve this problem in the future.

HP06T07 significantly suppressed tumor growth on the right injected and left uninjected sites, suggesting that intratumoral injection of HP06T07 triggered systemic antitumor immune responses. This is consistent with previous reports in which CpG ODNs inhibited growth of tumors on both sides in CT26, MCA38, TSA, and A20 mouse models (Wang S. et al., 2016; Sagiv-Barfi et al., 2018). HP06T07 promoted the accumulation of T cells, B cells, NK cells, and pDCs. However, the exact and in-depth mechanism of the antitumor effect of HP06T07 was not identified in the present study. Thus, immune system responses and other mechanisms that might be involved in the treatment with HP06T07 still require elucidation by further investigations.

## Conclusions

We have demonstrated that the novel CpG-C ODN, HP06T07, significantly induces B-cell functions, and IFN-α, IL-6, and TNF-α secretion in human and mouse *in vitro*. In addition, intratumoral injection of HP06T07 suppressed tumor growth at both primary and distant sites of CT26 tumors. In the future, with more studies, HP06T07 may be an excellent candidate for cancer therapy when used as a monotherapy or co-therapy.

## Data Availability Statement

The raw data analyzed during the current study are available from the corresponding authors upon reasonable request.

## Ethics Statement

This study was carried out in accordance with the recommendations of institutional guidelines, the ethics committee of the First Hospital of Jilin University with written informed consent from all subjects. All subjects gave written informed consent in accordance with the Declaration of Helsinki. The protocol was approved by the ethics committee of the First Hospital of Jilin University, Changchun, China (approval no.: 2017-031).

## Author Contributions

TL, SZ, GZ, SL, and YQ performed the *in vitro* experiments, analyzed the data, and revised the manuscript. TL, JW, XL, and WY performed the *in vivo* experiments and analyzed the data. TL, JWC, YS, and JZ assisted in the writing of the discussion and revised the manuscript. Y-JL and JTC conceived the study, revised the data, and wrote the manuscript. All authors read and approved the final manuscript.

## Funding

This study was financially supported by the National Natural Science Foundation of China (Grant 81870152, 81571534); the National Major Scientific and Technological Special Project for “Significant New Drugs Development” during the Twelfth Five-year Plan Period (Grant 2013ZX09102032); the Key Scientific Project of Jilin Province (Grant 20140204024YY); the Scientific and Technological Developing Plan of Jilin Province (Grant 20180101097JC); the Jilin Provincial Key Laboratory of Biotherapy (20170622011JC); the Fundamental Research Funds for the Central Universities, and the Program for JLU Science and Technology Innovative Research Team (No. 2017TD-08); the 13th Five-Year Science and Technology Research and Planning Project of the Education Department of Jilin Province (JJKH20190043KJ).

## Conflict of Interest

Authors YS and JZ were employed by company Changchun Huapu Biotechnology Co., Ltd.

The remaining authors declare that the research was conducted in the absence of any commercial or financial relationships that could be construed as a potential conflict of interest.
