# Supplementary material for: A Novel C Type CpG Oligodeoxynucleotide Exhibits Immunostimulatory Activity In Vitro and Enhances Antitumor Effect In Vivo
Source: Front Pharmacol. 2020 Feb 6;11:8. doi: 10.3389/fphar.2020.00008 (PMC7015978; doi:10.3389/fphar.2020.00008)
Supplement: Supplementary file 1 [file DataSheet_1.docx]

**Materials and Methods**

**1.1** **Generation of human** **monocyte-derived dendritic cells (MDDCs)**

Human MDDCs were isolated and cultured following established methods (Bauer et al., 2001; Pernthaner, et al., 2012). Briefly, monocytes were isolated from PBMCs by positive selection using anti-CD14 antibodies (Biolegend, San Jose, CA, USA) conjugated to anti-bio microbeads (Miltenyi Biotec, Bergisch Gladbach, Germany) according to the manufacturer’s instructions (Miltenyi Biotec, Bergisch Gladbach, Germany). The purity of the CD14^+^ monocytes was determined using a flow cytometer (FACS Array; BD Biosciences, San Jose, USA) and was > 95% (Fig. S1A). The monocytes were cultured at 1 × 10^6^ cells/mL in RPMI-1640 (Corning, NY, USA) supplemented with 10% fetal bovine serum (FBS; Clark, USA), 1% penicillin/streptomycin (TransGen Biotech, Beijing, China), 100 ng/mL human granulocyte-macrophage colony-stimulating factor (GM-CSF), and 50 ng/mL interleukin (IL)-4 (PeproTech Inc., NJ, USA), at 37 °C in humidified air containing 5% CO_2_. The medium was changed every 2 days. On day 6, the immature MDDCs were verified using FACS. Consistent with previous studies, MDDCs expressed CD11c and HLA-DR, whereas CD14 and CD83 expression were low (Fig. S1B).

**1.2 RNA extraction and reverse transcriptase-PCR (RT-PCR)**

Total RNA was isolated using *EasyPure*^®^ RNA Kit (TransGen Biotech, Beijing, China), and cDNA was reverse-transcribed using *TransScript*^®^ First-Strand cDNA Synthesis SuperMix (TransGen Biotech, Beijing, China) according to the manufacturer’s instructions. PCR was performed at 94 °C for 30 s, 62 °C for 30 s, and 72 °C for 30 s for 35 cycles using 2×*EasyTaq*^®^ PCR SuperMix (+dye) (TransGen Biotech, Beijing, China). The following primers specific for PCR of mouse TLR9 (mTLR9) and GAPDH were used: TLR9 forward, 5′-CCGCAAGACTCTATTTGTGCTGG-3′, and TLR9 reverse, 5′-TGTCCCTAGTCAGGGCTGTACTCAG-3′; and GAPDH forward, 5′-TGCACCACCAACTGCTTAGC-3′, and GAPDH reverse, 5′-GGATGCAGGGATGATGTTCT-3′. PCR products were visualized by agarose gel electrophoresis.

**1.3 Western blotting**

For monitoring mTLR9 protein expression, cells were lysed with RIPA buffer (Cell Signaling Technology, Danvers, MA, USA) containing protease inhibitor cocktail (MedChemExpress, New Jersey, USA). The cell lysates were separated by SDS-PAGE and transferred onto a PVDF membrane (Millipore, Bedford, MA, USA). The membrane was then blocked and probed with primary anti-mouse TLR9 antibody (1:1000; R&D Systems, Minneapolis, MN, USA) overnight at 4 °C. Labeled proteins were visualized by the enhanced chemiluminescence method using HRP-coupled secondary antibodies (PerkinElmer, Waltham, MA, USA).

**1.4 Activation of** **mouse mTLR9**

HEK-Blue™-mTLR9 cells (InvivoGen, San Diego, CA, USA) were established by co-transfecting HEK293 cells with murine *TLR9* gene and an inducible secreted embryonic alkaline phosphatase (*SEAP*) reporter gene. The *SEAP* gene was placed under the control of the interferon (IFN)-β minimal promoter fused to five nuclear factor (NF)-κB and activator protein (AP-1)-binding sites. mTLR9 activation by CpG ODNs was tested by reporter gene expression. HEK-Blue™ Null1 cells is the parental cell line of HEK-Blue™ mTLR9 cells (InvivoGen, San Diego, CA, USA). In a typical procedure, 3 × 10^4^ HEK-Blue™-mTLR9 cells and HEK-Blue™ Null1 cells were cultured in Dulbecco’s modified Eagle’s medium (DMEM; Gibco, New York, US) supplemented with 10% FBS (Gibco, New York, US), 1% penicillin/streptomycin (TransGen Biotech, Beijing, China), 30 μg/mL blasticidin (not required by HEK-Blue™ Null1 cells), 100 μg/mL zeocin, and 100 μg/mL normocin (InvivoGen, San Diego, CA, USA) in 96-well flat-bottom plates. After 72 h, HP06T07, ODN 2395, negative control (GC) and positive control TNF-α were added, followed by another 24 h incubation. SEAP levels were quantified by developing supernatants with QuantiBlue™ substrate (InvivoGen, San Diego, CA, USA) for 10 min and reading the absorption at 620 nm, following manufacturer’s instructions.

**Supplementary Figures**

**
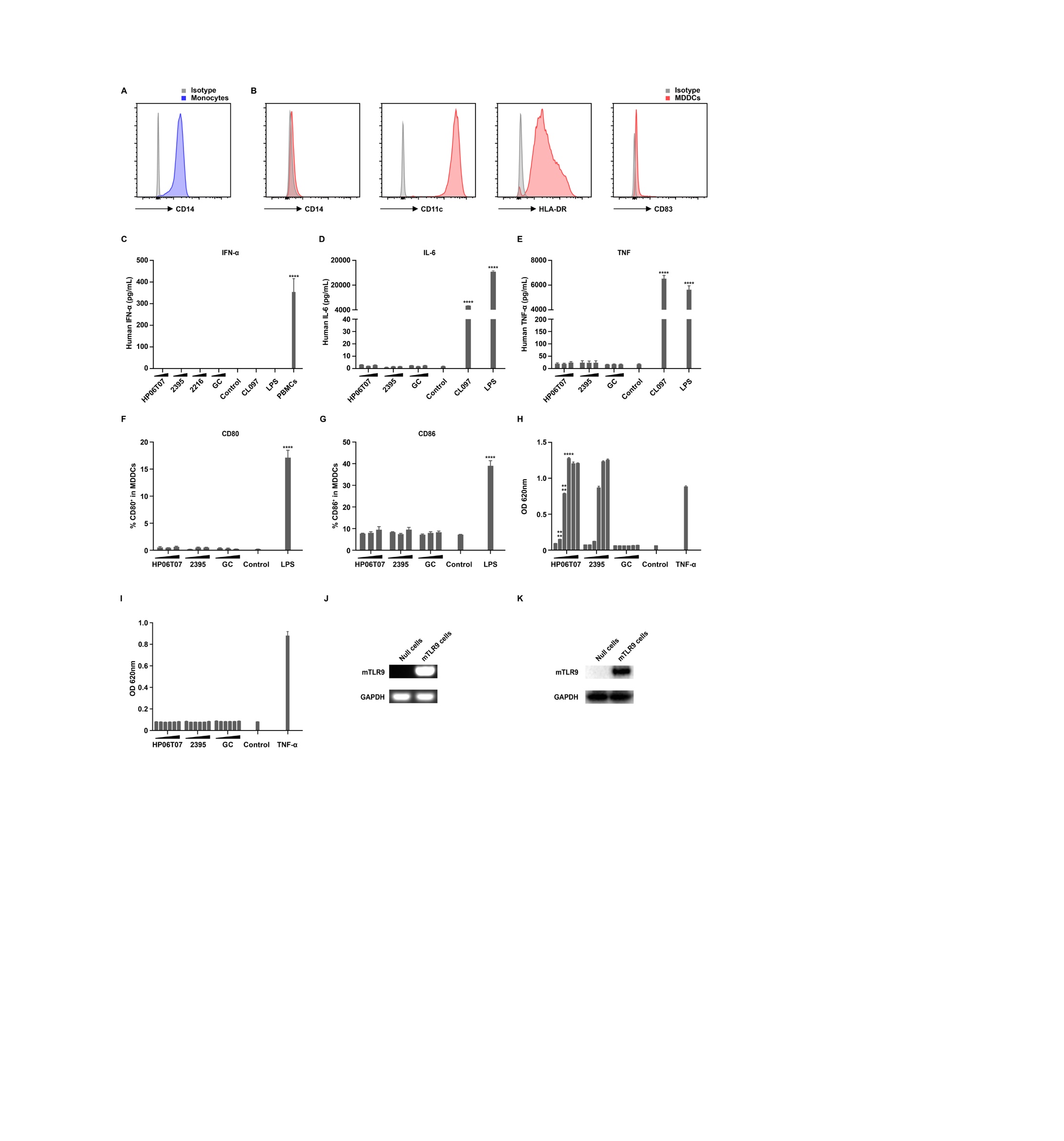
**

**Figure S1.** HP06T07 was specific to human and mouse TLR9. **(A)** The purity of CD14^+^ monocytes was detected using FACS. **(B)** Immature MDDCs was verified using FACS. **(C-G)** Immature MDDCs (2 × 10^5^) were cultured with or without HP06T07, ODN 2395, and GC at different concentrations (0.3, 1, and 3 μM) for 16 h. Supernatants were harvested and assayed for IFN-α **(C)** using ELISA, and IL-6 **(D)** and TNF **(E)** via CBA, and cells were harvested for staining with anti-human CD80 **(F)** and CD86 **(G)** antibodies. PBMCs stimulated with 1 μM HP06T07 were used as the positive control for IFN-α secretion, and MDDCs stimulated with ODN 2216 (0.3, 1, and 3 μM), CL097 (1 μg/mL) or lipopolysaccharide (LPS, 1 μg/mL) were used as the control for cytokine secretion, and for CD80 and CD86 expression. All data are means ± SEM (n = 5-6/group). Statistical significance of differences was determined (*P < 0.05, *P < 0.01, ***P < 0.001, and ****P < 0.0001). **(H-I)** Activation of mTLR9 stimulated by HP06T07, ODN2395, GC (0.01, 0.03, 0.1, 0.3, 1, and 3 μM) or TNF-α (10 ng/mL) in HEK-Blue™ mTLR9 cells **(H)** or HEK-Blue™ Null1 cells **(I)**. Data are means ± SEM (n = 6/group). Statistical significance of differences between HP06T07 and ODN 2395 groups were determined (*P < 0.05, *P < 0.01, ***P < 0.001, and ****P < 0.0001). **(J-K)** mTLR9 expression in HEK-Blue™ mTLR9 cells and HEK-Blue™ Null1 cells. Total RNA or protein was isolated, and RT-PCR **(J)** and Western blot analysis **(K)** were performed. The results are representatives of at least two independent experiments.

**References**

Bauer M., Redecke V., Ellwart J.W., Scherer B., Kremer J.P., Wagner H., et al. (2001). Bacterial CpG-DNA triggers activation and maturation of human CD11c^-^, CD123^+^ dendritic cells. J. Immunol. 166, 5000-5007.

Pernthaner A., Stasiuk S.J., Roberts J.M., Sutherland I.A. (2012). The response of monocyte derived dendritic cells following exposure to a nematode larval carbohydrate antigen. Vet. Immunol. Immunopathol. 148, 284-292.
